# Supplementary material for: A link between STK signalling and capsular polysaccharide synthesis in Streptococcus suis
Source: Nat Commun. 2023 Apr 29;14:2480. doi: 10.1038/s41467-023-38210-4 (PMC10148854; doi:10.1038/s41467-023-38210-4)
Supplement: Supplementary file 1 — Supplementary Information [file 41467_2023_38210_MOESM1_ESM.pdf]

Supplementary Information

A link between STK signalling and capsular polysaccharide synthesis in *Streptococcus suis*

Jinsheng Tang[1], Mengru Guo[1], Min Chen[1], Bin Xu[2], Tingting Ran[3], Weiwu Wang[3], Zhe Ma[1,4], Huixing Lin[1,4], Hongjie Fan[1,4,*]

[*]Corresponding author: Hongjie Fan, fhj@njau.edu.cn

This file includes:

Supplementary text
Figures S1 to S9
Tables S1 to S4
SI References

Supplementary figures

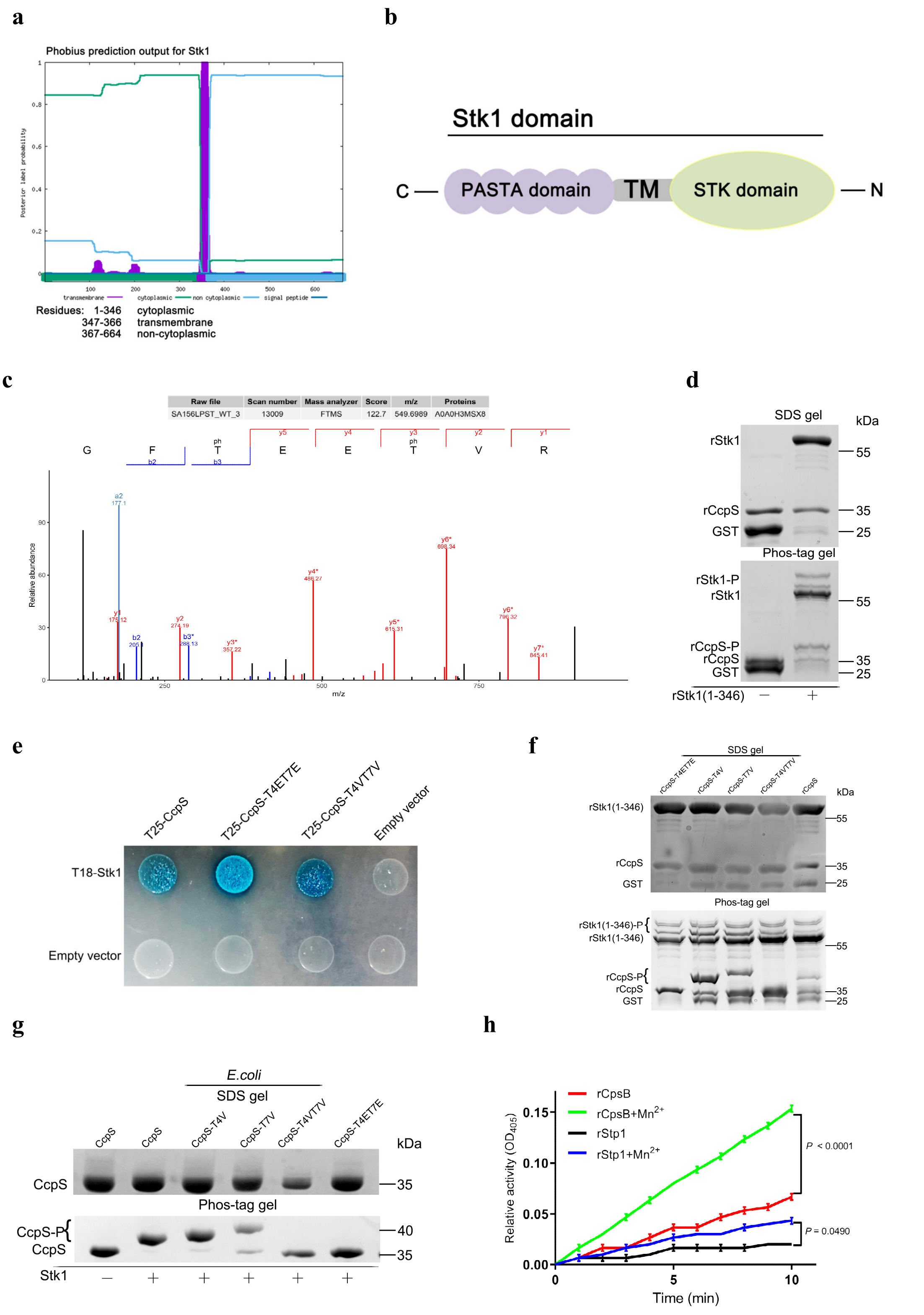

Supplementary Fig. 1 The CcpS phosphorylation catalyzed by the serine/threonine kinase Stk1. **a** The predicted transmembrane topology of Stk1 was shown, the Phobius can be found at (phobius.sbc.su.se). **b** The schematic shows the domain architecture of Stk1, including N-terminal STK kinase domain (1-346 amino acids) and the C-terminal PASTA domain (367-664 amino acids) separated by a transmembrane region (347-366 amino acids). **c** The phosphorylation sites of CcpS

were mapped by MS/MS spectrum. The b and y fragment ions were blue and red, respectively. And the neutral loss peak is marked with an asterisk. A neutral loss of phosphate (H_3PO_4-98Da) was detected in b3 to identify the phosphorylation site residue Thr4, and a neutral loss of phosphate (H_3PO_4-98Da) was detected in y3 to identify the phosphorylation site residue Thr7 of CcpS. **d, f** The phosphorylation of CcpS by Stk1 was analyzed in vitro. The recombinant truncated protein rStk1$_{1\text{-}346}$ phosphorylate rCcpS was detected by Phos-tag gel and a standard SDS gel, and Coomassie stain (**d**). The recombinant protein rCcpS variants also were analyzed (**f**). The position and identity of relevant bands is marked to the side. The experiment was repeated independently three times with similar results. **e** Bacterial two-hybrid assay testing for interactions between CcpS and its variants (CcpS-T4ET7E and CcpS-T4VT7V) with Stk1. Blue colony formation suggests that a direct interaction occurs. **g** rCcpS-GST and its variants were expressed in heterologous host *E. coli* in the absence or presence of Stk1, GST agarose beads used to pull down rCcpS-GST and the samples were loaded onto Phos-tag gel to analyze the phosphorylation of CcpS, samples also were loaded onto a standard SDS gel and Coomassie stain. The experiment was repeated independently three times with similar results. **h** The analysis of Stp1 and CpsB activity in vitro using para-nitrophenylphosphate (pNPP), which is a artificial substrate. The assay mixture contained 16 mM pNPP as a substrate in the absence or presence of 1 mM $MnCl_2$. Data represent mean ± SD from n = 3 biologically independent experiments. Statistical difference: two-way ANOVA followed by Tukey's post-tests. P values < 0.05 indicate significant differences. Source data are provided as a Source Data file.

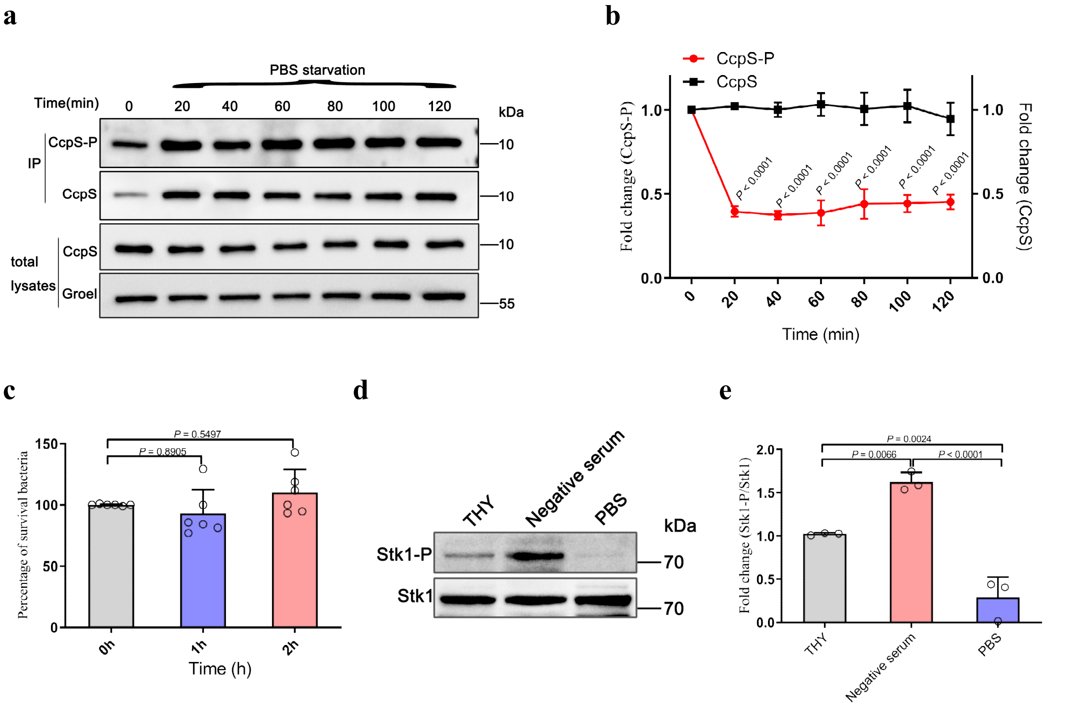

Supplementary Fig. 2 CcpS phosphorylation in *S. suis* is active, especially when cells against various stimuli. **a** IP analysis of *S. suis* CcpS using anti-CcpS beads from whole-cell lysates of cells with various treatments. Western blot of immunoprecipitate analysed on SDS gel using anti-CcpS antibodies for CcpS and anti-phosphothreonine antibody for CcpS-P. Exponentially growing cells were treated with starvation in PBS continuously, cells were harvested at the indicated time. **b** Bar graphs showed the levels of phosphorylated and total CcpS at different point time (Density analysis for **a**), and the values were normalized respectively to the 0 min levels, considered 1. Data represent mean ± SD from n = 3 biologically independent experiments. **c** Bar graphs showed that the survival bacterial cells. Cells against starvation in PBS, the survival cells were analyzed by plate THY agar plates at the point time. The values were normalized to the 0 h, considered 100%. Data represent mean ± SD from n = 6 biologically independent experiments. **d** IP analysis of *S. suis* Stk1 using anti-Stk1 beads from whole-cell lysates of cells with various treatments. Western blot of immunoprecipitate analysed on SDS gel using anti-Stk1 antibodies for Stk1 and anti-phosphothreonine antibody for Stk1-P. Exponentially growing cells were treated with starvation in PBS or nutrient medium (THY supplemented with negative serum for *S. suis* at ratio of 1:1) for 20 min. **e** Bar graphs showed the percentage of phosphorylated and total Stk1 in different treatment groups (Density analysis for **d**). Data represent mean ± SD from n = 3 biologically independent experiments. For **b, c, e**, Statistical difference: one-way ANOVA followed by Bonferroni or Tukey's post-tests. P values < 0.05 indicate significant differences. Source data are provided as a Source Data file.

a

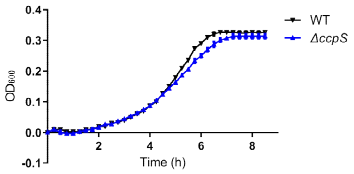

b

Drug name	MIC(μg/mL)					
	WT	*ΔccpS*	*ccpS-T4VT7V*	*ccpS-T4ET7E*	*Δstk1*	*Δstk1ΔccpS*
Ceftazidime (BS)	2.048	2.048	2.048	2.048	0.512	1.024
Ceftriaxone (BS)	0.256	0.256	0.256	0.256	0.128	0.128
Cefuroxime (ES)	0.256	0.256	0.256	0.256	0.064	0.128
Cefadroxil (NS)	0.512	0.512	0.512	0.512	0.128	0.256

BS, broad spectrum; ES, expanded spectrum; NS, narrow spectrum

c

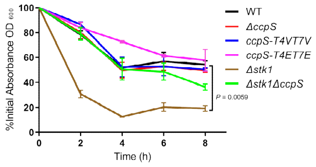

d

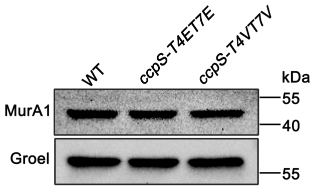

e

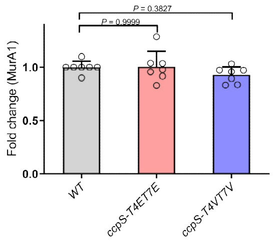

f

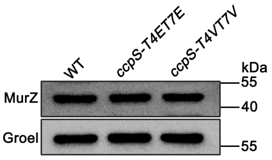

g

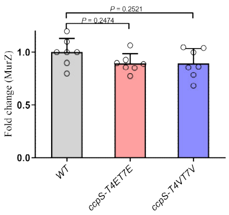

h

Drug name	MIC(μg/mL)			
	WT	*ΔccpS*	*ΔmurZ*	*ΔmurA1*
Ceftazidime (BS)	2.048	2.048	1.024	2.048
Ceftriaxone (BS)	0.256	0.256	0.256	0.256
Cefuroxime (ES)	0.256	0.256	0.256	0.256

BS, broad spectrum; ES, expanded spectrum; NS, narrow spectrum

i

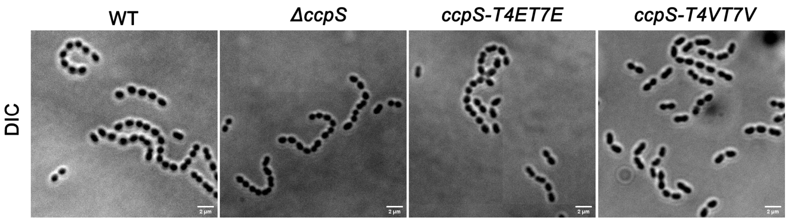

j

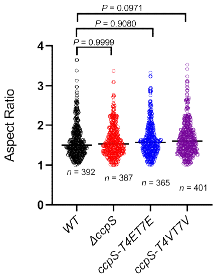

k

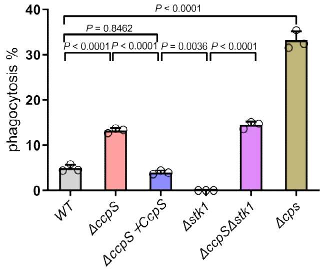

l

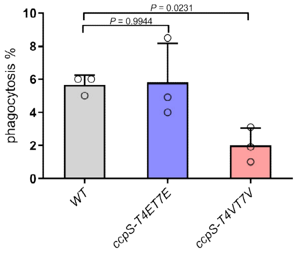

m

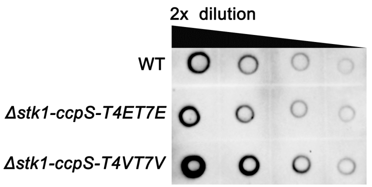

n

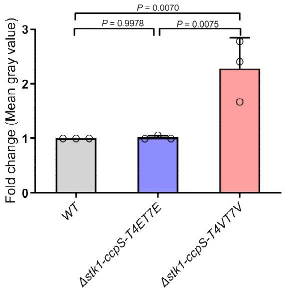

Supplementary Fig. 3 CcpS is required for resist phagocytosis and CPS synthesis in *S. suis*. **a** Effect of *ΔccpS* mutant on growth of *S. suis*. Growth of *S. suis* strain ZY05719 (WT) and *ΔccpS* in THY broth was recorded at 37°C. Indicated strains were grown overnight in THY and diluted into fresh THY medium to an OD_{600} = 0.02. Then the OD_{600} were read automatically every 15 min for 8 - 10 h. Growth experiments were performed three times and average values and standard deviations are shown. **b, h** Susceptibility analyses for antibiotics that target cell wall processes. MICs experiments performed in THY at 37°C for 24 h as described in Methods. **c** The rate of cells autolysis was determined as described in Methods. Data represent mean ± SD from *n* = 3 biologically independent experiments. Statistical difference: two-way ANOVA followed by Bonferroni's post-tests, compared to WT strain. **d, f** Western blot showing cellular protein levels of MurA1 (RS05565) (**d**) and MurZ (RS07665) (**f**) in the indicated strains. For this experiment, strains were grown in THY broth at 37˚C until an OD600 of 0.4-0.6 and the cells were collected. Groel served as loading controls. **e, g** Bar graphs showed the MurA1 and MurZ levels in different indicated strains (density analysis for **d** and **f**, respectively). The values were normalized to the WT levels, considered 1. Data represent mean ± SD from *n* = 7 biologically independent experiments. **i, j** Effect of CcpS and its phosphorylation on the overall cell shape of *S. suis*. Cells were grown in THY until OD600 reached 0.4-0.6. And samples were observed under the microscope. Aspect ratios were computed with MicrobeJ. *n* = 392 (WT), *n* = 387 (Δ*ccpS*), *n* = 365 (*ccpS-T4ET7E*), *n* = 401 (*ccpS-T4VT7V*). Three independent experiments were conducted. Bars represent the average aspect ratios. **k, l** Bacterial phagocytosis as described in Methods, the results are expressed as the percentage of CFU recovered bacteria/initial bacterial CFU. Data represent mean ± SD from *n* = 3 biologically independent experiments. **m** Dot blot showing serial dilutions (1:2) of spent growth media spotted on a PVDF membrane and probed with an anti-CPS antibodies. *S. suis* strain ZY05719 (WT), *Δstk1-ccpS-T4ET7E* (Strain expressing CcpS-T4ET7E in the *Δstk1* mutant strain background), *Δstk1-ccpS-T4VT7V* (Strain expressing CcpS-T4VT7V in the *Δstk1* mutant strain background) were grown in THY broth at 37˚C, cultures were normalized to an OD600 = 1.0 and same volume culture medium were assayed. **n** Bar graph showed the cell free CPS levels (Density analysis for **m**, at the second dilution). The values were normalized to the WT levels, considered 1. Data represent mean ± SD from *n* = 3 biologically independent experiments. For **e, g, j, k, l, n** Statistical difference: one-way ANOVA followed by Bonferroni or Tukey's post-tests. *P* values < 0.05 indicate significant differences. Source data are provided as a Source Data file.

a

Gene number	annotation	gene
RS07665	UDP-N-acetylglucosamine 1-carboxyvinyltransferase	*murZ*
RS05395	DUF4097 family beta strand repeat-containing protein	-
RS08535	MarR family transcriptional regulator	*marR*
RS08185	transcriptional regulator NrdR	*nrdR*
RS02785	tyrosine protein phosphatase	*cpsB*
RS02020	cell division regulator GpsB	*gpsB*
RS08485	acetyl-CoA carboxylase, carboxyltransferase subunit beta	*accD*

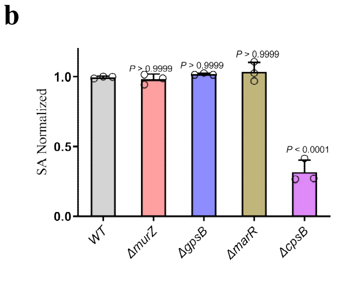

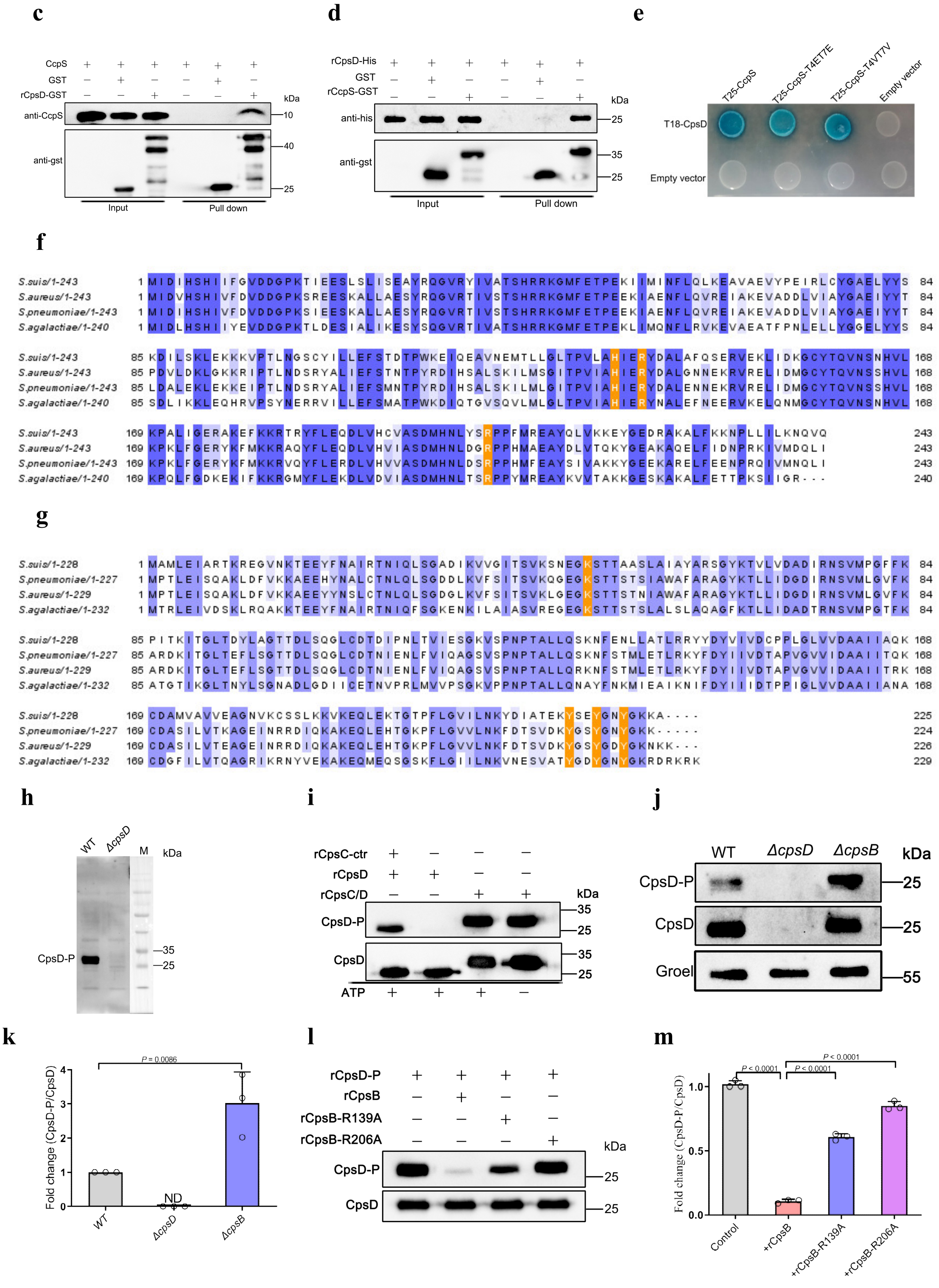

Supplementary Fig. 4 CcpS interacts directly with CpsB/CpsD，which are important parts of Wzx-Wzy pathway for CPS synthesis in bacteria. **a** Table listing the genes annotations of CcpS partners in *S. suis* strain ZY05719. **b** Bar graph showed the cell surface's sialic acid (SA) levels of *S. suis* strain ZY05719 (WT) and gene deletion strains. SA extracted from the cells' surface was quantified by a resorcinol assay. The values were normalized to the WT levels, considered 1. Data represent mean ± SD from *n* = 3 biologically independent experiments. **c-e** The interaction between CcpS and CpsD was detected in vitro and in vivo. Affinity purification of rCpsD-GST from *S. suis* whole-cell lysates pulls down native CcpS (**c**), Pull-down assay confirmed the direct interaction between CcpS and CpsD (**d**), and Bacterial two-hybrid assay testing for interactions between CcpS and CpsD (**e**). **f, g** Multiple sequence alignments of *S. suis* strain ZY05719 CpsB and CpsD protein sequences, conserved residues for potential catalytic sites in CpsB (**f**) or autokinase catalytic sites and tyrosine clusters in CpsD (**g**) were marked with orange. **h** Immunoblot showing the tyrosine phosphorylation patterns of the whole cells in *S. suis,* WT strain (left) and *ΔcpsD* (right) were probed with anti-phosphotyrosine antibodies. Images are representative of experiments performed in triplicate. **i** Autophosphorylation of CpsD in vitro. Recombinant proteins rCpsD incubated in presence or absence rCpsC-ctr at 37˚C with ATP, or chimera proteins rCpsC/D (means C-terminal sequence of CpsC fused into N-terminus of CpsD) were individual incubated in presence or absence ATP at 37˚C. Western blot of samples analysed on an SDS gel using anti-phosphotyrosine and anti-CpsD antibodies. Images are representative of experiments performed in triplicate. **j** Anti-phosphotyrosine immunoblot analysis of whole-cell lysates from the indicated strains. Immunoblot shows phosphorylated CpsD and total CpsD protein levels using anti-phosphotyrosine and anti-CpsD antibodies, respectively. Groel: loading control. **k** Bar graphs showed the percentage of phosphorylated and total CpsD in different groups (density analysis for **j**). The values were normalized to the WT levels, considered 1. Data represent mean ± SD from *n* = 3 biologically independent experiments. **l** De-phosphorylation of CpsD in vitro. Recombinant proteins rCpsD-P incubated in presence or absence of rCpsB and its variants (rCpsB-R139A and rCpsB-R206A) at 37˚C. Western blot of samples analysed on an SDS gel using anti-phosphotyrosine and anti-CpsD antibodies. **m** Bar graphs showed the percentage of phosphorylated and total CpsD in different groups (density analysis for **l**). The values were normalized to the control levels, considered 1. Data represent mean ± SD from *n* = 3 biologically independent experiments. For **b, k, m**, Statistical difference: one-way ANOVA followed by Bonferroni or Tukey's post-tests. ND, non-detected. *P* values < 0.05 indicate significant differences. Source data are provided as a Source Data file.

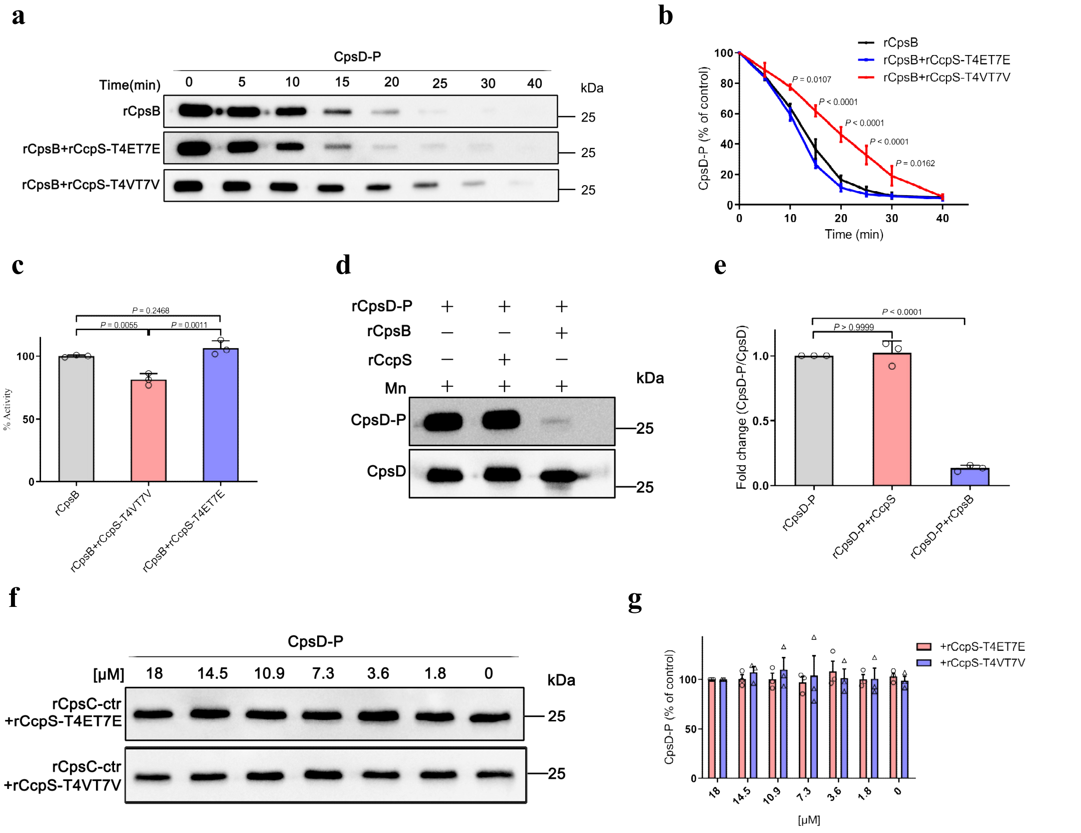

Supplementary Fig. 5 CcpS modulates the CpsB activity thereby altering CpsD phosphorylation, and the activity of CcpS is dependent on phosphorylation by kinase Stk1. **a** Phosphatase assays were performed as described in Methods, and the phosphorylated CpsD proteins were detected by SDS gel and Western blot using anti-phosphotyrosine antibodies. Recombinant rCcpS-T4ET7E or rCcpS-T4VT7V were pre-incubated with rCpsB, respectively, and then adding the rCpsD-P into the mixture to initiate the reaction. Immunoblot showing levels of CpsD phosphorylation using anti-phosphotyrosine antibodies at the indicated time-point in each group. **b** Bar graphs showed the phosphorylation levels of CpsD in each group at the indicated time-point (density analysis for **a**). The values were normalized to the 0 min levels in each group for each time-point, considered 100%. Data represent mean ± SD from n = 3 biologically independent experiments. Significant differences were determined by two-way ANOVA followed by Bonferroni's post-tests, compared to only rCpsB group. **c** Biochemical characterization of the activities of rCpsB in presence of rCcpS-T4ET7E or rCcpS-T4VT7V using pNPP, which is an artificial substrate. Each group rCpsB activity was normalized to the individual rCpsB incubation group, considered 100%. Data represent mean ± SD from n = 3 biologically independent experiments. **d** Effect of CcpS on CpsD-P in vitro. Recombinant proteins rCpsD-P incubated in presence or absence rCcpS and rCpsB at 37˚C with Mn^{2+} for 30 min. Western blot of samples analysed on an SDS gel using anti-phosphotyrosine and anti-CpsD antibodies. **e** Bar graphs showed the percentage of phosphorylated and total CpsD in different groups (density analysis for **d**). The values were normalized to the individual rCpsD-P incubation group levels, considered 1. Data represent mean ± SD from n = 3 biologically independent experiments. **f** Effect of CcpS variants (CcpS-T4ET7E and CcpS-T4VT7V) on auto-phosphorylation of CpsD in vitro. Recombinant proteins rCpsD incubated in presence or absence of different concentrations of rCcpS variants at 37˚C with ATP for 30 min. Western blot of samples analysed on an SDS gel using anti-phosphotyrosine. **g** Bar graphs showed the phosphorylation levels of CpsD in each group at the indicated concentration range (density analysis for **f**). The values were normalized to the 18 μM levels in each group for each concentration range, considered 100%, average values and standard deviations are calculated from three independent experiments. For **c**, **e**, **g** Statistical difference: one-way ANOVA followed by Bonferroni or Tukey's post-tests. P values < 0.05 indicate significant differences. Source data are provided as a Source Data file.

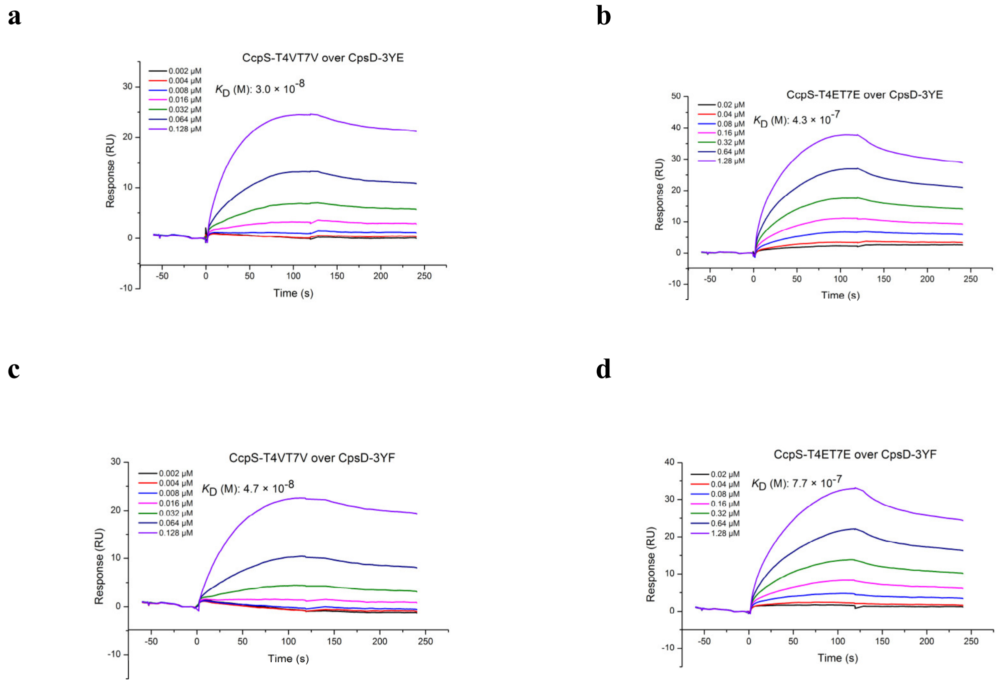

Supplementary Fig. 6 Phosphorylation of CcpS modulates the affinity to CpsD. **a-d** SPR analysis of the binding between CpsD and CcpS proteins. Gradient concentrations of the indicated proteins were flowed over immobilized CpsD-3YE or CpsD-3YF. Kinetic profiles are shown. CcpS-T4VT7V binding to CpsD-3YE (**a**). CcpS-T4ET7E binding to CpsD-3YE (**b**). CcpS-T4VT7V binding to CpsD-3YF (**c**). CcpS-T4ET7E binding to CpsD-3YF (**d**). Source data are provided as a Source Data file.

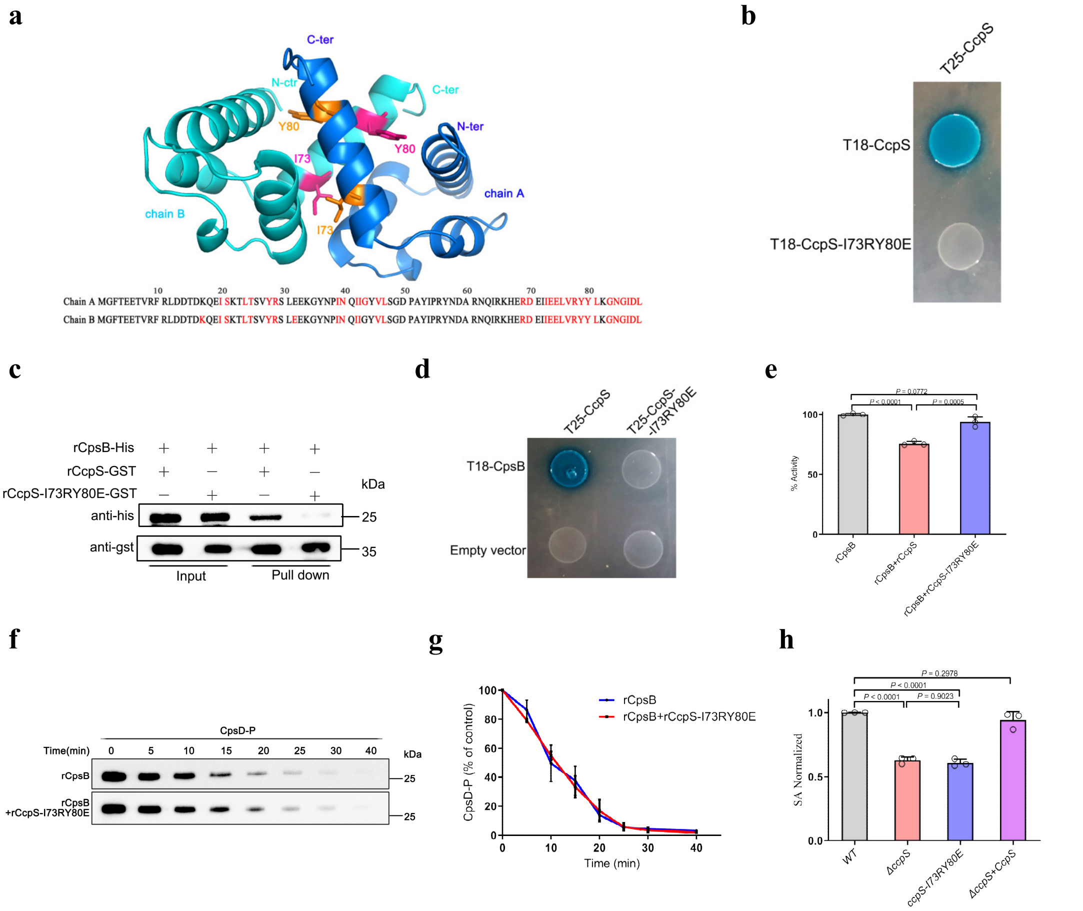

Supplementary Fig. 7 CcpS's homo-dimers is essential for its function in CPS synthesis modulation by regulating CpsB activity. **a** The structure of CcpS depicted as a cartoon with each protomer in the dimer coloured separately. The residues I73 and Y80 in the dimer interface were marked. And the bottom sequence indicated the interface residues were coloured with red, which may be involved in formation of dimer. **b, d** Bacterial two-hybrid assay testing for self-interactions of CcpS-I73RY80E variant (**b**) and its interaction with CpsB (**d**). Blue colony formation suggests that a direct interaction occurs. **c** Pull-down assay confirmed the interaction between CcpS, CcpS-I73RY80E variant and CpsB. Purified rCcpS-GST or rCcpS-I73RY80E-GST were incubated with rCpsB-His, and protein complexes were captured by GST agarose beads, washed, and eluted in sample buffer. Fractions were probed with anti-GST and anti-His antibodies. **e** Biochemical characterization of the activities of rCpsB in presence of rCcpS or rCcpS-I73RY80E variant using pNPP, which is a artificial substrate. Each group rCpsB activity was normalized to the individual rCpsB incubation group, considered 100%. Data represent mean ± SD from n = 3 biologically independent experiments. **f** Phosphatase assays were performed as described in Methods, and the phosphorylated CpsD proteins were detected by SDS gel and Western blot using anti-phosphotyrosine antibodies. Recombinant rCcpS or rCcpS-I73RY80E were pre-incubated with rCpsB, respectively, and then adding the rCpsD-P into the mixture to initiate the reaction. Immunoblot showing levels of CpsD phosphorylation using anti-phosphotyrosine antibodies in each group. **g** Bar graphs showed the phosphorylation levels of CpsD in each group at the indicated time-point (density analysis for **f**). The values were normalized to the 0 min levels in each group for each time-point, considered 100%. Data represent mean ± SD from n = 3 biologically independent experiments. Significant differences were determined by two-way ANOVA followed by Bonferroni's post-tests. **h** Bar graph showed the cell surface's sialic acid (SA) levels. SA extracted from the cells' surface was quantified by a resorcinol assay. The values were normalized to the WT levels, considered 1. Data represent mean ± SD from n = 3 biologically independent experiments. For **e, h**, Statistical difference: one-way ANOVA followed by Bonferroni or Tukey's post-tests. P values < 0.05 indicate significant differences. Source data are provided as a Source Data file.

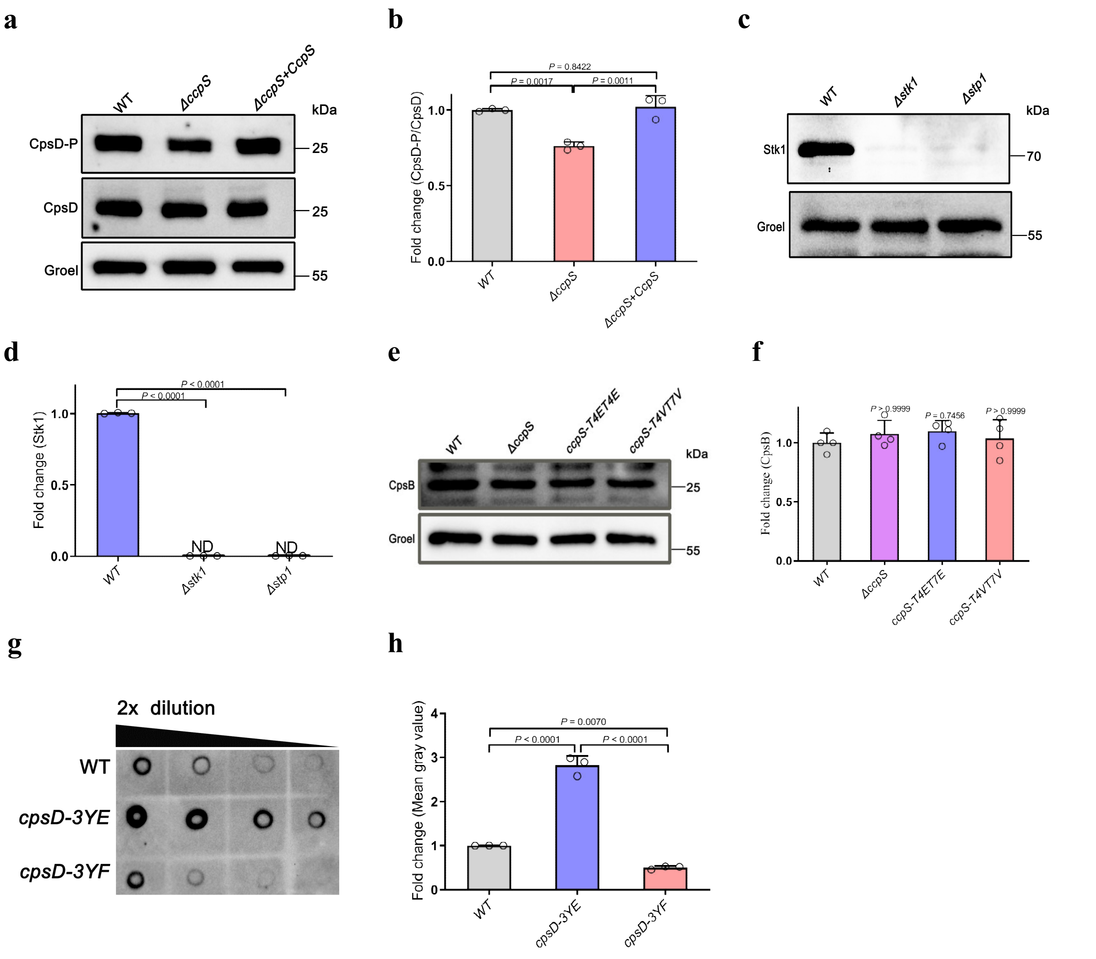

Supplementary Fig. 8 CcpS modulates CPS synthesis in *S. suis* by regulating CpsD phosphorylation. **a** Anti-phosphotyrosine immunoblot analysis of whole-cell lysates from the indicated strains. Immunoblot shows phosphorylated CpsD and total CpsD protein levels using anti-phosphotyrosine and anti-CpsD antibodies, respectively. Groel: loading control. **b** Bar graphs showed the percentage of phosphorylated and total CpsD in different groups (density analysis for **a**). The values were normalized to the WT levels, considered 1. Data represent mean ± SD from n = 3 biologically independent experiments. **c** Western blot showing cellular Stk1 levels in the indicated strains. Western blot of whole-cell lysates analysed on an SDS gel using anti-Stk1 antibodies. Groel served as loading controls. **d** Bar graphs showed the Stk1 levels in different indicated strains (density analysis for **c**). The values were normalized to the WT levels, considered 1. Data represent mean ± SD from n = 3 biologically independent experiments. ND, non-detected. **e** Western blot showing cellular levels of CpsB in the indicated strains. Western blot of whole-cell lysates analysed on an SDS gel using anti-CpsB antibodies. Groel served as loading controls. **f** Bar graphs showed the CpsB levels in different indicated strains (density analysis for **e**). The values were normalized to the WT levels, considered 1. Data represent mean ± SD from n = 4 biologically independent experiments. **g** Dot blot showing serial dilutions (1:2) of spent growth media spotted on a PVDF membrane and probed with an anti-CPS antibodies. **h** Bar graph showed the cell free CPS levels (Density analysis for **g**, at the second dilution). The values were normalized to the WT levels, considered 1. Data represent mean ± SD from n = 3 biologically independent experiments. For **b, d, f, h**, Statistical difference: one-way ANOVA followed by Bonferroni or Tukey's post-tests. P values < 0.05 indicate significant differences. Source data are provided as a Source Data file.

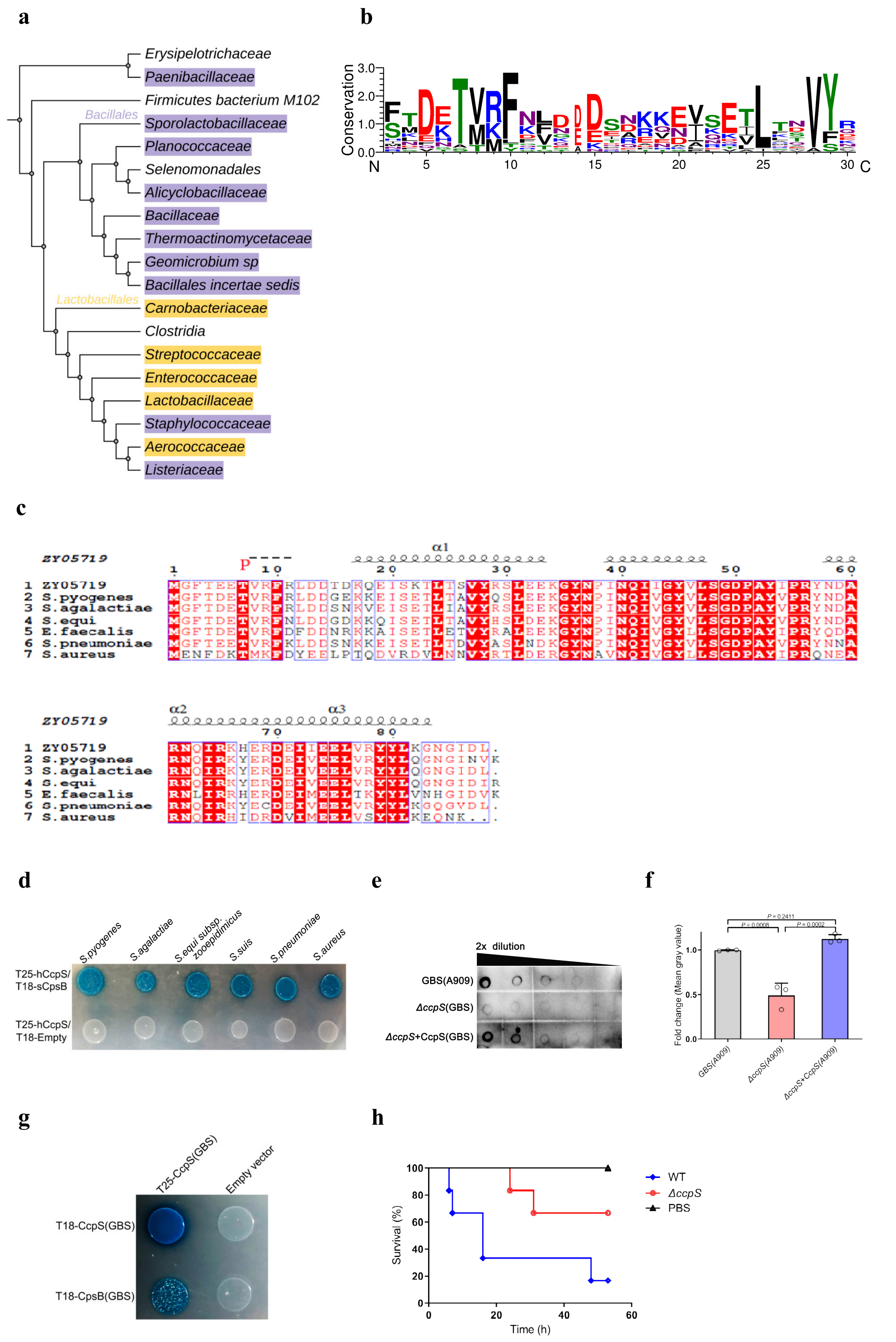

Supplementary Fig. 9 CcpS phosphorylation is conserved as well as its function to modulate CPS synthesis and probably the virulence potential in Gram-positive bacteria.

a CcpS conservation in Firmicutes. The phylogenetic tree was determined as described in Methods. Orange represent *Lactobacillales* and

purple represent *Bacillales*. **b** Sequence logo plot of aligned residues from CcpS sequence 1-30 amino acids, and the logo was generated using WebLogo3. **c** Multiple sequence alignment of the CcpS sequence. The conserved residues and phosphorylation site Thr7 were marked. **d, g** Bacterial two-hybrid assay testing for interactions of CcpS homologs (hCcpS) from other cocci with CpsB from *S. suis* (sCpsB)(**d**), interactions between *S. agalactiae* A909 CcpS(GBS) and CpsB(GBS)(**g**). **e** Dot blot showing the cells' CPS released to medium of *S. agalactiae* WT strain and its derivative strains. **f** Bar graph showed the cell free CPS levels (Density analysis for **e**, at the second dilution). The values were normalized to the WT levels, considered 1. Data represent mean ± SD from n = 3 biologically independent experiments. Statistical difference: one-way ANOVA followed by Tukey's post-tests. P values < 0.05 indicate significant differences. **h** Survival of BALB/c mice inoculated intraperitoneally with ~10^8 colony-forming units of WT ZY05719 strain and *ΔccpS* mutant strain (n = 6 mice per group). Statistical difference: Log-rank (Mantel–Cox) test (P = 0.0554) and Gehan–Breslow–Wilcoxon test (P = 0.0415), compared WT strain and *ΔccpS* mutant strain animals. Source data are provided as a Source Data file.

Supplementary tables

Table S1. Strain list

**Strains**	**Description/genotype**	**References**
Streptococcus suis ZY05719	Clinical isolate, wild-type	Lab stocks
Streptococcus agalactiae A909	Clinical isolate, wild-type	[1]
Streptococcus pyogenes ATCC 19615	Clinical isolate, wild-type	[2]
Streptococcus pneumoniae D39	Clinical isolate, wild-type	[3]
Streptococcus equi subsp. zooepidemicus ATCC 35246	Clinical isolate, wild-type	[4]
Staphylococcus aureus JE2	Clinical isolate, wild-type	[5]
Sl1	ZY05719, *Δstk1*	This work
Sl2	ZY05719, *ΔccpS*	This work
Sl3	ZY05719, *ΔgpsB*	This work
Sl4	ZY05719, *ΔmarR*	This work
Sl5	ZY05719, *ΔcpsB*	This work
Sl6	ZY05719, *ΔcpsD*	This work
Sl7	ZY05719, *ΔmurZ*	This work
Sl8	ZY05719, *Δstk1ΔccpS*	This work
Sl9	ZY05719, *Δstp1*	This work
Sl10	ZY05719, *ΔccpS*+CcpS(ectopic expression of CcpS by pSET2)	This work
Sl11	ZY05719, *ccpS-T4ET7E*	This work
Sl12	ZY05719, *ccpS-T4VT7V*	This work
Sl13	ZY05719, *ΔmurA1*	This work
Sl14	ZY05719, *ΔcpsBΔccpS*	This work
Sl15	ZY05719, *ΔcpsBccpS-T4VT7V*	This work
Sl16	ZY05719, *ΔcpsBccpS-T4ET7E*	This work
Sl17	ZY05719, *Δstk1ccpS-T4VT7V*	This work
Sl18	ZY05719, *Δstk1ccpS-T4ET7E*	This work
Sl19	ZY05719, *ΔcpsB*+CpsB(ectopic expression of CpsB by pSET2)	This work
Sl20	ZY05719, *ΔcpsD*+CpsD(ectopic expression of CpsD by pSET2)	This work
Sl21	ZY05719, *cpsB-R139A*	This work
Sl22	ZY05719, *cpsB-R206A*	This work
Sl23	ZY05719, *cpsD-3YE*	This work
Sl24	ZY05719, *cpsD-3YF*	This work
Sl25	ZY05719, *cpsD*+CpsD-K49A(ectopic expression of CpsD-K49A by pSET2)	This work
Sl26	ZY05719, *ccpS-I73RY80E*	This work
Sl27	ZY05719,*ΔccpS*+CcpS-T4ET7E(GBS)(ectopic expression of homologous CcpS from GBS)	This work
Sl28	ZY05719,*ΔccpS*+CcpS-T4VT7V(GBS)(ectopic	This work

	expression of homologous CcpS from GBS)	
Sl29	ZY05719,*ΔcpsEF*	This work
Sl30	A909,*ΔccpS*	This work
Sl31	A909,*ΔccpS*+CcpS	This work
Sl32	A909,*ΔcpsEF*	This work
E. coli DH5α	F- φ80dlacZΔM15 Δ(lacZYA-argF)U169 deoR recA1 endA1 hsdR17(rk-， mk+) phoA supE44 λ- thi-1 gyrA96 relA1	Lab stocks
E. coli BL21(DE3)	F- ompT hsdSB(rB-mB-) gal dcm(DE3)	Lab stocks
E. coli BTH101	F-, cya-99, araD139, galE15,galK16, rpsL1 (StrR), hsdR2,mcrA1, mcrB1, relA1	[6]

Table S1. List of strains used in this study.

Table S2. Plasmid list

Plasmids	Description/genotype	References
pSET4s	Suicide vector for gene deletions and insertions	[7]
pSET2	Construction of complementary strain	[8]
pGEX-4T-1	ampR protein expression vector	Clontech
pET-28a	kanR protein expression vector	Clontech
pUT18C	Plac::T18	[6]
pUT18	Plac::T18	[6]
pKT25	Plac::T25	[6]
pKNT25	Plac::T25	[6]
plS1	Plasmid for the deletion of stk1	This work
plS2	Plasmid for the deletion of ccpS	This work
plS3	Plasmid for the deletion of cpsB	This work
plS4	Plasmid for the deletion of cpsD	This work
plS5	Plasmid for the deletion of gpsB	This work
plS6	Plasmid for the deletion of murZ	This work
plS7	Plasmid for the deletion of marR	This work
plS8	Plasmid for the deletion of cpsEF	This work
plS9	Plasmid for the deletion of stp1	This work
plS10	Plasmid as template for CcpS point mutation	This work
plS11	Plasmid for replacing the native ccpS locus with T4ET7E	This work
plS12	Plasmid for replacing the native ccpS locus with T4VT7V	This work
plS19	Plasmid for replacing the native ccpS locus with T4VT7VI73RY80E	This work
plS20	Plasmid as template for cpsB point mutation	This work
plS21	Plasmid for replacing the native cpsB locus with R139A	This work
plS22	Plasmid for replacing the native cpsB locus with R206A	This work
plS23	Plasmid as template for cpsD point mutation	This work
plS24	Plasmid for replacing the native cpsD locus with 3YE	This work
plS25	Plasmid for replacing the native cpsD locus with 3YF	This work
plS26	ZY05719 cpsD with native promoter cloned into pSET2	This work
plS27	ZY05719 ccpS with native promoter cloned into pSET2	This work
plS28	ZY05719 cpsB with native promoter cloned into pSET2	This work
plS29	ZY05719 cpsD carrying mutation K49A in pSET2	This work
plS30	ccpS from ZY05719 in pET-28a	This work
plS31	ccpS from ZY05719 carrying mutation T4ET7E in pET-28a	This work
plS32	ccpS from ZY05719 carrying mutation T4VT7V in pET-28a	This work
plS33	ccpS from ZY05719 carrying mutation I73RY80E in pET-28a	This work
plS34	cpsB from ZY05719 in pET-28a	This work
plS35	cpsB from ZY05719 carrying mutation R139A in pET-28a	This work
plS36	cpsB from ZY05719 carrying mutation R206A in pET-28a	This work
plS37	cpsD from ZY05719 in pET-28a	This work
plS38	cpsD from ZY05719 carrying mutation 3YE in pET-28a	This work
plS39	cpsD from ZY05719 carrying mutation 3YF in pET-28a	This work

plS40	cpsD from ZY05719 carrying mutation K49A in pET-28a	This work
plS41	ccpS17-88AA from ZY05719 in pGEX-4T-1	This work
plS42	cpsC201-229AA from ZY05719 in pGEX-4T-1	This work
plS43	chimera cpsC/D from ZY05719 in pET-28a	This work
plS44	Stk1 from ZY05719 in pGEX-4T-1	This work
plS45	Stp1 from ZY05719 in pET-28a	This work
plS46	Stk1 1-346AA from ZY05719 in pET-28a	This work
plS47	ccpS17-88AA from ZY05719 in pET-28a	This work
plS48	ccpS from ATCC 19615 in pGEX-4T-1	This work
plS49	ccpS from A909 in pGEX-4T-1	This work
plS50	ccpS from ATCC 35246 in pGEX-4T-1	This work
plS51	ccpS from ZY05719 in pGEX-4T-1	This work
plS52	ccpS from D39 in pGEX-4T-1	This work
plS53	ccpS from JE2 in pGEX-4T-1	This work
plS54	ccpS from ZY05719 carrying mutation T4ET7E in pGEX-4T-1	This work
plS55	ccpS from ZY05719 carrying mutation T4VT7V in pGEX-4T-1	This work
plS56	Plasmid for the deletion of hccpS (homologous *ccpS* from GBS)	This work
plS57	Plasmid for the deletion of hcpsEF (homologous *cpsEF* from GBS)	This work
plS58	A909 ccpS with native promoter cloned into pSET2	This work
plS59	ccpS from A909 carrying mutation T4ET7E in pSET2	This work
plS60	ccpS from A909 carrying mutation T4VT7V in pSET2	This work
plS61	Plac::t25-ccpS	This work
plS62	Plac::t25-stk1	This work
plS63	Plac::t25-ccpS-T4ET7E	This work
plS64	Plac::t25-ccpS-T4VT7V	This work
plS65	Plac::t25-ccpS from ATCC 19615	This work
plS66	Plac::t25-ccpS from A909	This work
plS67	Plac::t25-ccpS from ATCC 35246	This work
plS68	Plac::t25-ccpS from D39	This work
plS69	Plac::t25-ccpS from JE2	This work
plS70	Plac::t18-ccpS	This work
plS71	Plac::t18-stk1	This work
plS72	Plac::t18-cpsB	This work
plS73	Plac::t18-cpsD	This work
plS74	Plac::t18-ccpS from ATCC 19615	This work
plS75	Plac::t18-ccpS from A909	This work
plS76	Plac::t18-ccpS from ATCC 35246	This work
plS77	Plac::t18-ccpS from D39	This work
plS78	Plac::t18-ccpS from JE2	This work
plS79	Plac::t18-ccpS-T4ET7E	This work

plS80	Plac::t18-ccpS-T4VT7V	This work
plS81	Plac::t18-cpsB from A909	This work
plS82	Plac::ccpS-t25	This work

Table S2. List of plasmids used in this study.

Table S3. Primer list

Primer name	Sequence
ol1	ACGCGTCGACTCATCGGTCAACTTAGACGAAAATC
ol2	CCAACCTTATTCATCTTTATTACCGATTTGAATCATTACC
ol3	GGTAATGATTCAAATCGGTAATAAAGATGAATAAGGTTGG
ol4	CGCGGATCCCTTTTTGAACCTGTAGAGAATAGTC
ol5	TAAAACGACGGCCAGTGAATTC TTACCCCAAAATTTAAAGTT
ol6	GTCAATCCCATTCCCTTTCAA CATACTTACACCTTCTTTCC
ol7	GGAAAGAAGGTGTAAGTATG TTGAAAGGGAATGGGATTGAC
ol8	GCAGGTCGACTCTAGAGGATCC TCTCCGTTCTCATCTTCTTC
ol9	TAAAACGACGGCCAGTGAATTC AATAGGAGAACAATATGGCG
ol10	CACTATTTCTATGAGGTTA CATATTAATACCTACTTATC
ol11	GATAAGTAGGTATTAATATG TAACCTCATAGAAATAGTG
ol12	GCAGGTCGACTCTAGAGGATCC AAACCGAATGAATTAATATC
ol13	TAAAACGACGGCCAGTGAATTC AAGGCAGCGATTCAGTCCGT
ol14	GCTTTTTTGCCGTAATTTCC TTCTAACATCGCCATATTG
ol15	CAATATGGCGATGTTAGAA GGAAATTACGGCAAAAAAGC
ol16	GCAGGTCGACTCTAGAGGATCC GCCTTCGCTCTATCCTCACC
ol17	TAAAACGACGGCCAGTGAATTC AGGACTGGTTCTATTG
ol18	GTCAGTTTAGGTGTACAATTT AATACTTGCCATCTCTCTCTC
ol19	GAGAGAGAGATGGCAAGTATT AAATTGTACACCTAAACTGAC
ol20	GCAGGTCGACTCTAGAGGATCC GAACTTGCAGCCTAGAG
ol21	TAAAACGACGGCCAGTGAATTC GCCAGAACATCTCCAGCACG
ol22	GAATGTTAGTATGAGAAAAA TGATTGAGGATTAGAGAGTC
ol23	GACTCTCTAATCCTCAATCA TTTTTCTCATACTAACATTC
ol24	GCAGGTCGACTCTAGAGGATCC TGCTTTGGTTGATAGAGTGG
ol25	TAAAACGACGGCCAGTGAATTC ATACGTATATTGGCTTGGTG
ol26	AAGTAAAGGAGGCCTGTT CATCAACGATTTCACAATG
ol27	CATTGTGAAATCGTTGATG AACAGGCCTCCTTTACTT
ol28	GCAGGTCGACTCTAGAGGATCC GAAAGGAGCAAGAGAATGGC
ol29	TAAAACGACGGCCAGTGAATTC AACATAAGTTTGATAAGTAG
ol30	CTTCTCATACTCATCAACAA TTCAATATTCATAGCTCCTC
ol31	GAGGAGCTATGAATATTGAA TTGTTGATGAGTATGAGAAG
ol32	GCAGGTCGACTCTAGAGGATCC TTTCTAATTCTACTACTCGC
ol33	TAAAACGACGGCCAGTGAATTCAAGTGGAGCGATTGGCAGAG
ol34	CTCCGTAATGTGAAGCAAACCAATTTCCATAATTTTTATC
ol35	GATAAAAATTATGGAAATTGGTTTGCTTCACATTACGGAG
ol36	GCAGGTCGACTCTAGAGGATCCTTTGCCGTTGCTTTTAGTAC
ol37	CGCGGATCC GTATCTAGTTGTACTTCTT
ol38	ACGCGTCGAC GAATCTCCTTTAAAATG
ol39	GGGATTTGAAGAAGAAGAAGTTCGTTTTCGCCTAG
ol40	AAACGAACTTCTTCTTCTTCAAATCCCATACTTACAC
ol41	GATTTGTCGAAGAAGTCGTTCGTTTTCGCCTAG
ol42	AACGACTTCTTCGACAAATCCCATACTTACAC

ol43	GATTTGTCGAAGAAACCGTTCGTTTTCGCCTAG
ol44	AACGGTTTCTTCGACAAATCCCATACTTACAC
ol45	GATTTACCGAAGAAGTCGTTCGTTTTCGCCTAG
ol46	AACGACTTCTTCGGTAAATCCCATACTTACAC
ol47	GGGATTTGAAGAAGAAgtcGTTCGTTTTCGCCTAG
ol48	AAACGAACgacTTCTTCTTCAAATCCCATACTTACAC
ol49	GATTTGTCGAAGAAGAAGTTCGTTTTCGCCTAG
ol50	AACTTCTTCTTCGACAAATCCCATACTTACAC
ol51	GATTTGCCGAAGAAGCCGTTCGTTTTCGCCTAG
ol52	AACGGCTTCTTCGGCAAATCCCATACTTACAC
ol53	GGGATTTGACGAAGAAGACGTTCGTTTTCGCCTAG
ol54	AAACGAACGTCTTCTTCGTCAAATCCCATACTTACAC
ol55	GAATTGGTGCGCTACGAGTTGAAAGGG
ol56	GCGCACCAATTCTTCTCTGATTTCATC
ol57	TAAAACGACGGCCAGTGAATTCAGATATTAAGGTTGTTGGTATTACCTC
ol58	GCAGGTCGACTCTAGAGGATCCTCATTATGATAAAAATTCCAGAACG
ol59	TATAGAGGCTTATGATGCTCTGGCATTTCAG
ol60	ATCATAAGCCTCTATATGGGCAAGTACGGG
ol61	TATAGTGCACCTCCGTTTATGAGGGAGGCGT
ol62	CGGAGGTGCACTATATAAATTATGCATATCG
ol63	TAAAACGACGGCCAGTGAATTC TATTTGGCAAAAGACTATC
ol64	GCAGGTCGACTCTAGAGGATCC CCCAAGTAGCGTCATTTC
ol65	GAGAAGGAGAGTGAAGAGGGAAATGAGGGCAAAAAAGCCTAATTT
ol66	TTTGCCCTCATTTCCCTCTTCACTCTCCTTCTCAGTGGCAATAT
ol67	AGAAGTTTAGTGAATTCGGAAATTTCGGCAAAAAAGCCTAATTT
ol68	TTGCCGAAATTTCCGAATTCACTAAACTTCTCAGTGGCAATAT
ol69	TGAAGGTGCGAGTACAACTGCGGCTAGTC
ol70	TTGTACTCGCACCTTCATTCGATTTAAC
ol71	TAAAACGACGGCCAGTGAATTC CTTGCGAAGGAAGCCATTAC
ol72	GCAGGTCGACTCTAGAGGATCC ATCAACTGGAAGTTTGTAAC
ol73	TAAAACGACGGCCAGTGAATTC AAAAAAAGGCAGAGGAAGAT
ol74	GCAATTTCTAACATCGCCAT AAACAACCTTCCTAGACTT
ol75	AAGTCTAGGAAGGTTGTTT ATGGCGATGTTAGAAATTGC
ol76	GCAGGTCGACTCTAGAGGATCC ATGGATATCAATCATATTAATAC
ol77	TAAAACGACGGCCAGTGAATTC AAAAAAAGGCAGAGGAAGAT
ol78	CGGTGTGCCTGTTTTTTC AAACAACCTTCCTAGACTT
ol79	AAGTCTAGGAAGGTTGTTT GAAAAAACAGGCACACCG
ol80	GCAGGTCGACTCTAGAGGATCC CTATGAGGTTACTGTACTTG
ol81	GCAAATGGGTCGCGGATCC GAGAATCTTTATTTTCAGGGCGCC ATGGGATTTACCGAAG
ol82	TGTCGACGGAGCTCGAATTC TTAGAGGTCAATCCCATTCC
ol83	GGATTTGAAGAAGAAGAAGTTCGTTTTCGCCTAGATG

ol84	AACGAACTTCTTCTTCTTCAAATCCCATGGCGCCCTG
ol85	GATTTGTCGAAGAAGTCGTTCGTTTTCGCCTAG
ol86	ACGAACGACTTCTTCGACAAATCCCATGGCGCCCTG
ol87	GAATTGGTGCGCTACGAGTTGAAAGGG
ol88	GCGCACCAATTCTTCTCTGATTTCATC
ol89	AAGAAGGAGATATACCATGGGC ATGATTGATATCCATTCGC
ol90	TGTCGACGGAGCTCGAATTC TTAATGGTGATGGTGATGATG CTGTACTTGATTTTTC
ol91	TATAGAGGCTTATGATGCTCTGGCATTTCAG
ol92	ATCATAAGCCTCTATATGGGCAAGTACGGG
ol93	TATAGTGCACCTCCGTTTATGAGGGAGGCGT
ol94	CGGAGGTGCACTATATAAATTATGCATATCG
ol95	AGCAAATGGGTCGCGGATCC ATGGCGATGTTAGAAATTG
ol96	TGTCGACGGAGCTCGAATTC TTAGGCTTTTTTGCCGT
ol97	GAGAAGGAGAGTGAAGAGGGAAATGAGGGCAAAAAAGCCTAATTT
ol98	TTTGCCCTCATTTCCCTCTTCACTCTCCTTCTCAGTGGCAATAT
ol99	AGAAGTTTAGTGAATTCGGAAATTTCGGCAAAAAAGCCTAATTT
ol100	TTGCCGAAATTTCCGAATTCACTAAACTTCTCAGTGGCAATAT
ol101	TGAAGGTGCGAGTACAACTGCGGCTAGTC
ol102	TTGTACTCGCACCTTCATTCGATTTAAC
ol103	TCCAGGGGCCCCTGGGATCC AAACAAGAAATCAGCAAGAC
ol104	TCGAGTCGACCCGGGAATTC TTAGAGGTCAATCCCATTCC
ol105	TCCAGGGGCCCCTGGGATCC GATGACCGTGTAAAACGTCC
ol106	TCGAGTCGACCCGGGAATTC CTATTTTAATTTCTTCGAATC
ol107	AGCAAATGGGTCGCGGATCC TTGGATGACCGTGTAAAACGTCC
ol108	CAATTTCTAACATCGCCAT TTTTAATTTCTTCGAATC
ol109	GATTCGAAGAAATTAAAA ATGGCGATGTTAGAAATTG
ol110	TGTCGACGGAGCTCGAATTC TTAGGCTTTTTTGCCGT
ol111	AGCAAATGGGTCGCGGATCC ATGATTCAAATCGGTAAG
ol112	TGTCGACGGAGCTCGAATTC TTATTGTCCGCTACCTG
ol113	AGCAAATGGGTCGCGGATCC ATGGAAATTGCATTACTT
ol114	TGTCGACGGAGCTCGAATTC TTACCTAGCCTCCTCCGT
ol115	AGCAAATGGGTCGC GGATCC ATGATTCAAATCGGT
ol116	GCGGCCGCAAGCTT GTCGAC TTA TTTGTAGCGTGTACGTATG
ol117	GCAAATGGGTCGCGGATCC GAGAATCTTTATTTTCAGGGCGCC AAACAAGAAATCAGCA
ol118	TGTCGACGGAGCTCGAATTC TTAGAGGTCAATCCCATTCC
ol119	TCGAGTCGACCCGG GAATTC TCATTTAACATCAATTCC
ol120	TCCAGGGGCCCCTG GGATCC ATGGGATTTACAGATGAAAC
ol121	TCGAGTCGACCCGGGAATTC TTACAAATCTATTCCATTTC
ol122	TCCAGGGGCCCCTGGGATCC ATGGGATTTACAGATGAAAC
ol123	TCGAGTCGACCCGGGAATTC TCATCTAATATCTATTCCAT
ol124	TCCAGGGGCCCCTGGGATCC ATGGGATTTACAGATGAAAC
ol125	CGCGGATCC ATGGGATTTACCGAAGAAAC

ol126	ACGCGTCGAC TTAGAGGTCAATCCCATTCC
ol127	TCCAGGGGCCCCTG GGATCC ATGGGATTTACTGAAG
ol128	TCGAGTCGACCCGG GAATTC TTATAGATCGACTCCTTGT
ol129	TCGAGTCGACCCGG GAATTC TTATTTATTTTGCTCTTTT
ol130	TCCAGGGGCCCCTG GGATCC ATGGAAAACTTTGATAAAAC
ol131	GGATTTGAAGAAGAAGAAGTTCGTTTTCGCCTAGATG
ol132	CGAACTTCTTCTTCTTCAAATCCCATGGATCCCAGGG
ol133	GGATTTGTCGAAGAAGTCGTTCGTTTTCGCCTAGATG
ol134	CGAACGACTTCTTCGACAAATCCCATGGATCCCAGGG
ol135	TAAAACGACGGCCAGTGAATTC TCTGTAAATGAGTAAGCTTG
ol136	GGAAAGAAGGTGTAGGTATG AATGGAATAGATTTGTAATG
ol137	CATTACAAATCTATTCCATT CATACCTACACCTTCTTTCC
ol138	GCAGGTCGACTCTAGAGGATCC GGTCTTAAGTCCCATGTTTG
ol139	TAAAACGACGGCCAGTGAATTC GCATTCATAAACGTCGCTGG
ol140	GAAAGAAAAAGAAAATATAC GGTGCTAAGTAAAGGTAAGG
ol141	CCTTACCTTTACTTAGCACC GTATATTTTCTTTTTCTTTC
ol142	GCAGGTCGACTCTAGAGGATCC GTTTAGAAATAGTTGATAGC
ol143	TAAAACGACGGCCAGTGAATTC GATATTCAACTGGAAGATTG
ol144	GCAGGTCGACTCTAGAGGATCC GTTATGCTAAGGCGCTCAAT
ol145	AACTTCTTCATCTTCAAATCCCATACCTACACCTT
ol146	TTTGAAGATGAAGAAGTTCGTTTTAGATTAGATG
ol147	AACGACTTCATCGACAAATCCCATACCTACACCTT
ol148	TTTGTCGATGAAGTCGTTCGTTTTAGATTAGATG
T25-CcpS-F	GCGCGCACGCGGCGGGCTGCAG GG ATGGGATTTACCGAAGAAAC
T25-CcpS-R	TTACTTAGGTACCCGGGGATCC TC TTAGAGGTCAATCCCATTCC
T25-Stk1-F	GCGCGCACGCGGCGGGCTGCAG GG ATGATTCAAATCGGT
T25-Stk1-R	TTACTTAGGTACCCGGGGATCC TC TTTGTAGCGTGTACGTATG
T25-ccpS-T4ET7E-F	GGATTTGAAGAAGAAGAAGTTCGTTTTCGCCTAGATG
T25-ccpS-T4ET7E-R	CGAAAACGAACTTCTTCTTCTTCAAATCCCAT
T25-ccpS-T4VT7V-F	GATTTGTCGAAGAAGTCGTTCGTTTTCGCCTAG
T25-ccpS-T4VT7V-R	CGAAAACGAACGACTTCTTCGACAAATCCCAT
T25-GAS-CcpS-F	TTACTTAGGTACCCGGGGATCC TC TCATTTAACATCAATTCC
T25-GAS-CcpS-R	GCGCGCACGCGGCGGGCTGCAG GG ATGGGATTTACAGATGAAAC
T25-GBS-CcpS-F	TTACTTAGGTACCCGGGGATCC TC TTACAAATCTATTCCATTTC
T25-GBS-CcpS-R	GCGCGCACGCGGCGGGCTGCAG GG ATGGGATTTACAGATGAAAC
T25-SEZ-CcpS-F	TTACTTAGGTACCCGGGGATCC TC TCATCTAATATCTATTCCAT
T25-SEZ-CcpS-R	GCGCGCACGCGGCGGGCTGCAG GG ATGGGATTTACAGATGAAAC
T25-D39-CcpS-F	GCGCGCACGCGGCGGGCTGCAG GG ATGGGATTTACTGAAG
T25-D39-CcpS-R	TTACTTAGGTACCCGGGGATCC TC TTATAGATCGACTCCTTGT
T25-JE2-CcpS-F	TTACTTAGGTACCCGGGGATCC TC TTATTTATTTTGCTCTTTT
T25-JE2-CcpS-R	GCGCGCACGCGGCGGGCTGCAG GG ATGGAAAACTTTGATAAAAC
T18-CcpS-F	GCAGGTCGACTCTAGAGGATCC C ATGGGATTTACCGAAGAAAC
T18-CcpS-R	ACTTAGTTATATCGATGAATTC GA TTAGAGGTCAATCCCATTCC

T18-Stk1-F	GCAGGTCGACTCTAGAGGATCC C ATGATTCAAATCGGT
T18-Stk1-R	ACTTAGTTATATCGATGAATTC GA TTATTGTCCGCTACCTG
T18-cpsB-F	GCAGGTCGACTCTAGAGGATCC C ATGGCGATGTTAGAAATTG
T18-cpsB-R	ACTTAGTTATATCGATGAATTC GA TTAGGCTTTTTTGCCGT
T18-cpsD-F	GCAGGTCGACTCTAGAGGATCC C ATGATTGATATCCATTCGC
T18-cpsD-R	ACTTAGTTATATCGATGAATTC GA TTACTGTACTTGATTTTTCAAT
T18-GAS-CcpS-F	ACTTAGTTATATCGATGAATTC GA TCATTTAACATCAATTCC
T18-GAS-CcpS-R	GCAGGTCGACTCTAGAGGATCC C ATGGGATTTACAGATGAAAC
T18-GBS-CcpS-F	ACTTAGTTATATCGATGAATTC GA TTACAAATCTATTCCATTTC
T18-GBS-CcpS-R	GCAGGTCGACTCTAGAGGATCC C ATGGGATTTACAGATGAAAC
T18-SEZ-CcpS-F	ACTTAGTTATATCGATGAATTC GA TCATCTAATATCTATTCCAT
T18-SEZ-CcpS-R	GCAGGTCGACTCTAGAGGATCC C ATGGGATTTACAGATGAAAC
T18-D39-CcpS-F	GCAGGTCGACTCTAGAGGATCC C ATGGGATTTACTGAAG
T18-D39-CcpS-R	ACTTAGTTATATCGATGAATTC GA TTATAGATCGACTCCTTGT
T18-JE2-CcpS-F	ACTTAGTTATATCGATGAATTC GA TTATTTATTTTGCTCTTTT
T18-JE2-CcpS-R	GCAGGTCGACTCTAGAGGATCC C ATGGAAAACTTTGATAAAAC
CcpS-T25-F	GCAGGTCGACTCTAGAGGATCC C ATGGGATTTACCGAAGAAAC
CcpS-T25-R	CATTGAATTCGAGCTCGGTACC CG GAGGTCAATCCCATTCC

Table S3. List of primers used in this study.

Table S4. Crystal structures data collection and refinement statistics

parameter	CcpS	CcpS-T4ET7E
**Data collection**		
Wavelength (Å)	0.97918	0.97918
Temperature (K)	100	100
Crystal-to-detector distance (mm)	177	177
Rotation range per image (o)	0.5	0.5
Total rotation range (o)	360	360
Space group	P21 21 21	P21 21 21
Unit-cell parameters (Å)	a = 41.34, b = 48.61, c = 84.51 β = 90.0°	a = 44.96, b = 49.08, c = 102.58, β = 90.0°
Resolution range (Å)	42.13-1.6 (1.657-1.6)	28.06-1.5 (1.554-1.5)
Observed reflections	258664 (19098)	407642 (14063)
Unique reflections	23141 (2270)	36796 (3350)
Multiplicity	6.7 (6.7)	11.1 (5.8)
Rpim (%)	0.036 (0.889)	0.036 (0.773)
Completeness (%)	99.87 (99.65)	98.95 (90.88)
I/σ(I)	12.1 (1.1)	9.9 (0.9)
**Structure refinement**		
Total number of atoms	1235	1286
No. of reflections used	23117 (2262)	36761 (3328)
Rwork(%)	0.2040 (0.3378)	0.2182 (0.3025)
Rfree(%)	0.2303 (0.3805)	0.2320 (0.3129)
Rmsd bonds (Å)	0.006	0.008
Rmsd angles (o)	0.73	1.21
Ramachandran plot (%)		
Favored	100	99.32
Allowed	0	0.68
Outlier	0	0

Statistics for the highest-resolution shell are shown in parentheses.

Supplementary References

1. Tettelin, H. et al. Genome analysis of multiple pathogenic isolates of *Streptococcus agalactiae*: Implications for the microbial "pan-genome". *P Natl Acad Sci USA* **102**, 13950-13955 (2005).
2. Minogue, T.D. et al. Complete Genome Assembly of *Streptococcus pyogenes* ATCC 19615, a Group A β-Hemolytic Reference Strain. *Genome Announc* **2** (2014).
3. Lanie, J.A. et al. Genome sequence of Avery's virulent serotype 2 strain D39 of *Streptococcus pneumoniae* and comparison with that of unencapsulated laboratory strain R6. *J Bacteriol* **189**, 38-51 (2007).
4. Ma, Z. et al. Complete Genome Sequence of *Streptococcus equi subsp zooepidemicus* Strain ATCC 35246. *J Bacteriol* **193**, 5583-5584 (2011).
5. Fey, P.D. et al. A genetic resource for rapid and comprehensive phenotype screening of nonessential *Staphylococcus aureus* genes. *Mbio* **4**, e00537-e00512 (2013).
6. Karimova, G., Pidoux, J., Ullmann, A. & Ladant, D. A bacterial two-hybrid system based on a reconstituted signal transduction pathway. *P Natl Acad Sci USA* **95**, 5752-5756 (1998).
7. Takamatsu, D., Osaki, M. & Sekizaki, T. Thermosensitive suicide vectors for gene replacement in *Streptococcus suis*. *Plasmid* **46**, 140-148 (2001).
8. Takamatsu, D., Osaki, M. & Sekizaki, T. Construction and characterization of *Streptococcus suis-Escherichia coli* shuttle cloning vectors. *Plasmid* **45**, 101-113 (2001).
