# Supplementary material for: A link between STK signalling and capsular polysaccharide synthesis in Streptococcus suis
Source: Nat Commun. 2023 Apr 29;14:2480. doi: 10.1038/s41467-023-38210-4 (PMC10148854; doi:10.1038/s41467-023-38210-4)
Supplement: Supplementary file 3 — Reporting Summary [file 41467_2023_38210_MOESM3_ESM.pdf]

nature portfolio

Corresponding author(s): Hongjie Fan
Last updated by author(s): Mar 27, 2023

Reporting Summary

Nature Portfolio wishes to improve the reproducibility of the work that we publish. This form provides structure for consistency and transparency in reporting. For further information on Nature Portfolio policies, see our Editorial Policies and the Editorial Policy Checklist.

Statistics

For all statistical analyses, confirm that the following items are present in the figure legend, table legend, main text, or Methods section.

n/a	Confirmed	
☐	☒	The exact sample size (*n*) for each experimental group/condition, given as a discrete number and unit of measurement
☐	☒	A statement on whether measurements were taken from distinct samples or whether the same sample was measured repeatedly
☐	☒	The statistical test(s) used AND whether they are one- or two-sided<br>*Only common tests should be described solely by name; describe more complex techniques in the Methods section.*
☒	☐	A description of all covariates tested
☒	☐	A description of any assumptions or corrections, such as tests of normality and adjustment for multiple comparisons
☐	☒	A full description of the statistical parameters including central tendency (e.g. means) or other basic estimates (e.g. regression coefficient) AND variation (e.g. standard deviation) or associated estimates of uncertainty (e.g. confidence intervals)
☐	☒	For null hypothesis testing, the test statistic (e.g. *F*, *t*, *r*) with confidence intervals, effect sizes, degrees of freedom and *P* value noted<br>*Give P values as exact values whenever suitable.*
☒	☐	For Bayesian analysis, information on the choice of priors and Markov chain Monte Carlo settings
☒	☐	For hierarchical and complex designs, identification of the appropriate level for tests and full reporting of outcomes
☒	☐	Estimates of effect sizes (e.g. Cohen's *d*, Pearson's *r*), indicating how they were calculated

Our web collection on statistics for biologists contains articles on many of the points above.

Software and code

Policy information about availability of computer code

Data collection	The following softwares were used for data collection: i-control 3.8.2.0 software (Tecan); ChemiDocTM Touch Imaging System(Bio-Rad); BioSpectrometer(eppendorf);Microscopy images were acquired using HT7800(Hitachi);The beamline BL19U1 of Shanghai Synchrotron Radiation Facility (SSRF); Zeiss AxioObserver Z1 microscope fitted with an Orca-R2 C10600 charge-coupled device (CCD) camera (Hamamatsu); Biacore T200 system , NanoDrop spectrophotometer (Thermo Fisher Scientific)
Data analysis	Clustal Omega 1.2.3 and Jalview were used for multiple-sequence alignments and presentation<br>Online PSI-BLAST (E-value < 10) was used for protein searches<br>mega 7.0 was used for building phylogenetic tree<br>iTOL 4.2.3 (online) was used for phylogenetic tree visualization<br>PyMOL v2.2 was used for protein structure visualization<br>MaxQuant v 1.5.3.17) was used for label-free quantification<br>Phobius(phobius.sbc.su.se) was used for transmembrane prediction<br>CLUSTALW(genome.jp/tools-bin/clustalw) was ued for multiple-sequence alignments<br>PSIPRED 4.0 (bioinf.cs.ucl.ac.uk/psipred) secondary structure analysis<br>ESPript 3.0 was used for presentation<br>ImageJ v 1.4<br>MicrobeJ (http://www.microbej.com/index.html) (Ducret et al. 2016, Nat.Microbiol. 1, 16077)<br>PHENIX V1.11.1-2575: https://www.phenix-online.org/<br>Coot v0.8.1: https://www2.mrc-lmb.cam.ac.uk/personal/pemsley/coot/<br>GraphPad Prism version 8.2.1.441 software

Biacore T200 Evaluation Software v 3.0, 2.0
REFMAC5 v 5.5.0026
Microsoft PowerPoint 2010(ScienceSlides) for model schematics
XDS Version January 10, 2022
Phaser-2.7.17
Discovery Studio v3.5
AutoDock Vina v1.1.2
WebLogo 3 (weblogo.threeplusone.com)
CLUSTALW (genome.jp/tools-bin/clustalw)
origin v 8.5
Photoshop CS6 v 13.0.1

For manuscripts utilizing custom algorithms or software that are central to the research but not yet described in published literature, software must be made available to editors and reviewers. We strongly encourage code deposition in a community repository (e.g. GitHub). See the Nature Portfolio guidelines for submitting code & software for further information.

Data

Policy information about availability of data

All manuscripts must include a data availability statement. This statement should provide the following information, where applicable:

- Accession codes, unique identifiers, or web links for publicly available datasets
- A description of any restrictions on data availability
- For clinical datasets or third party data, please ensure that the statement adheres to our policy

S. suis ZY05719 genome can be found at National Center for Biotechnology Information with the accession number of GI: 820722437 (https://www.ncbi.nlm.nih.gov/nuccore/820722437). The structure related data of CcpS and its mutant of S. suis generated in this study have been deposited in the RCSB Protein Data Bank under accession code: 7Y8Z (https://www.rcsb.org/structure/unreleased/7Y8Z) and 7Y86 (https://www.rcsb.org/structure/unreleased/7Y86), respectively. The data supporting the findings of this study are available in this article and its Supplementary files, or from the corresponding authors upon request. Source data are provided with this paper.

Human research participants

Policy information about studies involving human research participants and Sex and Gender in Research.

Reporting on sex and gender	N/A
Population characteristics	N/A
Recruitment	N/A
Ethics oversight	N/A

Note that full information on the approval of the study protocol must also be provided in the manuscript.

Field-specific reporting

Please select the one below that is the best fit for your research. If you are not sure, read the appropriate sections before making your selection.

☒ Life sciences ☐ Behavioural & social sciences ☐ Ecological, evolutionary & environmental sciences

For a reference copy of the document with all sections, see nature.com/documents/nr-reporting-summary-flat.pdf

Life sciences study design

All studies must disclose on these points even when the disclosure is negative.

Sample size	No sample-size calculations were performed. Sample sizes were chosen to allow appropriate statistical tests and the sample size was also determined according to our experience and were in line with other published studies in the field. (e.g.,Cleverley RM, Rutter ZJ, Rismondo J, et al. Nat Commun. 2019;10(1):261.; Choi S, Choi E, Cho YJ, Nam D, Lee J, Lee EJ. Nat Commun. 2019;10(1):3326. ; Horstmann N, Saldaña M, Sahasrabhojane P, et al. PLoS Pathog. 2014;10(5):e1004088. ; Schwechheimer C, Hebert K, Tripathi S, et al. PLoS Pathog.2020;16 (8):e1008745.;Toniolo C, Balducci E, Romano MR, et al. J Biol Chem. 2015;290(15):9521-9532. Nakamoto R, Kwan JMC, Chin JFL, et al. Proc Natl Acad Sci U S A. 2021;118(45):e2103377118.
Data exclusions	No data was excluded from the analyses.
Replication	All attempts at replicating the results were successful.Experiments were performed at least three biological replicates
Randomization	For mice were infected with S. suis randomly and allocated into different groups. Other samples, e.g., the bacteria culture samples and

	biochemical samples were maintained under the same environment and also allocated randomly into different groups. Samples were allocated in groups according to their genetic background and same treatment used when required.
Blinding	Mouse virulence assay were performed by an experimenter who was blinder to samples. Enumeration of the bacterial CFUs in the cell infection samples were also performed blindly. Investigators were not blinded during data collection or analysis since there was not group allocation. Other experiments were not blindly performed since the results could be directly obtained from visual observations or by instrument measurements.

Reporting for specific materials, systems and methods

We require information from authors about some types of materials, experimental systems and methods used in many studies. Here, indicate whether each material, system or method listed is relevant to your study. If you are not sure if a list item applies to your research, read the appropriate section before selecting a response.

Materials & experimental systems

n/a	Involved in the study
☐	☒ Antibodies
☐	☒ Eukaryotic cell lines
☒	☐ Palaeontology and archaeology
☐	☒ Animals and other organisms
☒	☐ Clinical data
☒	☐ Dual use research of concern

Methods

n/a	Involved in the study
☒	☐ ChIP-seq
☒	☐ Flow cytometry
☒	☐ MRI-based neuroimaging

Antibodies

Antibodies used	Anti-His tag mouse mAb(Engibody AT0025) ;<br>Anti-GST-Tag mouse mAb(Engibody AT0027) ;<br>Goat-anti-mouse IgG(H+L)-HRP(Engibody AT0098) ;<br>Goat anti-Rabbit IgG(H+L) (HRP)(Engibody AT0097);<br>Anti-CcpS, Stk1,CpsD, Groel, MurZ, MurA1,and CpsB IgG from mouse serum;<br>Rabbit anti-Phospho-Threonine Antibody (P-Thr-Polyclonal) (Cell Signaling 9381S);<br>Mouse anti-Phosphotyrosine antibody [PY20] (abcam ab10321);<br>Serum anti-serotype 2 from mouse;<br>goat anti-mouse IgG conjugated with Alexa Fluor 647
Validation	All commercial antibodies (AT0025,AT0027,AT0098,AT0097,9381S,ab10321, etc) were purchased from providers who have validated the antibodies for the use of Western blot.Antibody, catalogue number, manufacturer information for commercial antibodies:<br>Anti-His tag mouse mAb(Engibody, AT0025) https://www.engibody.com/products/His-tag-mouse-mab-Epitope-Tag-Antibody-at0025.html;<br>Anti-GST-Tag mouse mAb(Engibody ,AT0027)https://www.engibody.com/products/GST-tag-mouse-mab-Epitope-Tag-Antibody-at0027.html ;<br>Goat-anti-mouse IgG(H+L)-HRP(Engibody, AT0098)https://www.engibody.com/products/Goat-Anti-Mouse-IgG-H-L-HRP-AT0098.html ;<br>Goat anti-Rabbit IgG(H+L) (HRP)(Engibody , AT0097)https://www.engibody.com/products/Goat-Anti-Rabbit-IgG-H-L-HRP-AT0097.html;<br>Rabbit anti-Phospho-Threonine Antibody (P-Thr-Polyclonal) (Cell Signaling , 9381S)https://www.cellsignal.com/products/primary-antibodies/phospho-threonine-tyrosine-antibody/9381?site-search-type=Products&N=4294956287&Ntt=+9381s&fromPage=plp&_requestid=1112792;<br>Mouse anti-Phosphotyrosine antibody [PY20] (abcam, ab10321)https://www.abcam.cn/phosphotyrosine-antibody-py20-ab10321/reviews/13753;<br>goat anti-mouse IgG conjugated with Alexa Fluor 647(abcam, ab150115)https://www.abcam.cn/products/secondary-antibodies/goat-mouse-igg-hl-alexa-fluor-647-ab150115.html;<br>All homemade serum anti-CcpS, Stk1,CpsD, Groel, and CpsB IgG, etc, were validated in the present study by Western blot using appropriate positive and negative controls in our lab.

Eukaryotic cell lines

Policy information about cell lines and Sex and Gender in Research

Cell line source(s)	Mouse macrophage cell line RAW264.7 cells obtained from ATCC
Authentication	RAW264.7 cells obtained from ATCC were certified authentic, authenticity was monitored based on morphology.
Mycoplasma contamination	RAW264.7 cells obtained from ATCC were certified mycoplasma free

Commonly misidentified lines (See ICLAC register)	No commonly misidentified lines were used.

Animals and other research organisms

Policy information about studies involving animals; ARRIVE guidelines recommended for reporting animal research, and Sex and Gender in Research

Laboratory animals	Six-week-old female BALB/c mice purchased from the Comparative Medicine Center of Yangzhou University (Yangzhou, China) were used in this study.
Wild animals	No wild animals were used in the study.
Reporting on sex	Female mice were used in this study because it is easier to keep the female mouse together. Sex was not considered in the study design.
Field-collected samples	No field-collected samples were used in the study.
Ethics oversight	All animal experiments were approved by the Laboratory Animal Welfare and Ethics Committee of Nanjing Agricultural University, China (approval number NJAU.No20210510065 and NJAU.No20211005144). The Chinese National Laboratory Animal Guideline for Ethical Review of Animal Welfare adhered to animal care and protocol.

Note that full information on the approval of the study protocol must also be provided in the manuscript.
